# Using model texts as a type of feedback in EFL writing

**DOI:** 10.3389/fpsyg.2023.1156553

**Published:** 2023-06-29

**Authors:** Zhixin Wu, Jiaxin Qie, Xuehua Wang

**Affiliations:** ^1^School of Foreign Languages, Southeast University, Nanjing, China; ^2^Hohhot No.2 High School, Hohhot, Inner Mongolia, China

**Keywords:** EFL writing, language analytic ability, written corrective feedback, argumentation, model texts

## Abstract

Recent work has established that model texts could be employed as a useful feedback technique. However, few studies have employed argumentative writing tasks and analyzed draft quality, and little is known about the role played by the language analytic ability in using model texts. The current study aimed to investigate what Chinese EFL learners (*n* = 60) noticed at the composition and comparison (comparing their texts with model texts) processes in a four-stage argumentative writing task and explore to what degree model texts can enhance the learners’ subsequent writing. The four stages were: (1) writing (pre-test); (2) comparing (treatment); (3) rewriting (immediate post-test); (4) delayed writing (delayed post-test). The findings showed that learners primarily noticed lexical features in the composition and comparison stages. Higher language analytic ability (LAA) learners and guided noticing learners could notice and elicit more information from the model texts. Overall, the use of model texts was effective in improving learners’ writing by providing alternative elements associated with lexis, grammar, content, and organization. In addition, the beneficial effect of model texts on writing could be maintained after 1 week. Some pedagogical implications are put forward to help teachers make better use of model texts to improve learners’ writing. This study also provides new insights into how language analytic ability affects the effectiveness of using models and provides more information on the type of learner most likely to benefit from model texts.

## 1. Introduction

The past 30 years have seen a growing trend towards written corrective feedback (WCF) in the domain of second language (L2) acquisition and writing. Feedback can encourage learners to concentrate on form-meaning mapping and help identify the gap between their current language competence and the desired one (Sachs and Polio, 2007). A considerable amount of literature has been published investigating the effectiveness of WCF. Growing empirical studies have shown that WCF may enhance learners’ writing accuracy (Bitchener, 2016; Benson and Dekeyser, 2019). A large part of the WCF research has examined the effectiveness of feedback as a function of its scope (focused versus comprehensive/unfocused feedback) and different feedback strategies, mainly negative evidence feedback, such as direct feedback, and indirect feedback (see Kang and Han, 2015 for a review). When teachers provide negative evidence feedback on L2 writing, they frequently attempt to mark as many errors as possible in the hope that their students could compose at least one writing without errors (Lee, 2011). From the perspective of students, feedback seems to be the act of teachers delivering information to them in order to deliver correct answers and identify different types of errors. Unexpectedly, the overabundance of correction could demotivate certain students (Lee, 2008).

More recently, a growing interest exists to explore positive evidence feedback like model texts (García Mayo and Labandibar, 2017; Ramón, 2019; Kang, 2020; Lazaro-Ibarrola, 2021; Kang, 2022). While negative evidence feedback mainly addresses lexical and grammatical errors, model texts can play a useful role in presenting various suggestions about alternative expressions, content, and organizational structures. These studies suggest that the model text as a type of WCF is a valid educational tool for facilitating L2 learners in their writing in actual instruction. This type of feedback provides a better balance between focus on form and focus on meaning, and demonstrates how the ideas could be conveyed in a target-like manner. However, most previous work has investigated the effect of model texts by employing a narrative story writing assignment (García Mayo and Labandibar, 2017; Ramón, 2019; Luquin and Mayo, 2021). In addition, the long-term effect of model texts has hardly been examined since most existing studies were completed in a brief period of time (Cao and Mao, 2022). To further uncover the effect of model texts on EFL writing, some studies explored the effect of learner factors, such as language proficiency (Coyle and Roca De Larios, 2014; Cánovas Guirao et al., 2015), learners’ attitudes towards models (García Mayo and Labandibar, 2017), and working memory (Kang, 2022). To date, little work has been done on the role of other individual and contextual elements in fostering or inhibiting learners’ involvement with model texts (Cao and Mao, 2022).

In an attempt to bridge these gaps and examine in more detail whether model texts can be used effectively as a WCF strategy, the present research examined the availability of multi-paragraph model texts in an argumentation assignment that was recognized as one critical type in academic research at various levels of education (Qin and Karabacak, 2010). Moreover, this study added a delayed post-test to reveal the long-term effect of model texts and investigated how LAA may contribute to the potential benefits of employing models as positive evidence feedback.

## 2. Literature review

The feasibility of adopting model texts as a feedback strategy in EFL writing could be understood from the perspective of noticing and output. Several studies have documented the importance of noticing (Schmidt, 1990) for L2 development. It has been stated that learners may be motivated to notice when they discover they do not know the appropriate forms to convey a certain meaning in the context of interaction. When learners devote attentional resources to specific aspects of the input, noticing may also occur. Schmidt emphasized the necessity of learners’ involvement while comparing the interlanguage (IL) generated by themselves with the target language (TL) came from the input. This aspect of noticing is also known as “noticing the gap” (Schmidt and Frota, 1986; Schmidt, 1990; Schmidt, 2001). Besides, noticing is an important cognitive term for describing feedback as an internal process (Qi and Lapkin, 2001). The emphasis is on the utilization of feedback to help students concentrate on specific aspects of their writing abilities. According to Hyland (2011), when L2 students were able to identify the discrepancy between their present writing competence and intended learning targets, they could review their writing processes. Such kind of repeated self-evaluation of writing performance might be helpful in internalizing the feedback. Several elements have been considered for fostering or limiting learners’ noticing, including external factors like input, and feedback, internal factors like learners’ aptitude and different affective factors (Robinson, 1995; Izumi, 2002; Izumi, 2003; Dörnyei, 2009; Mackey et al., 2010). Model texts can act as supplementary knowledge which may prompt learners to compare the present competence with expected learning objectives. In this way, their knowledge structures and output can be modified and revised. Therefore, it is possible that learners become aware of their linguistic inadequacies in the writing process and further discover a discrepancy between the language usage of their composition and that of the models (Izumi, 2003; Hanaoka, 2007; Hanaoka and Izumi, 2012). On this point, model texts could be adopted as a type of corrective feedback.

Using model texts to deliver feedback for L2 learners’ errors is a comparatively under-explored strategy in WCF studies. Model texts refer to native-like texts that are adapted to “the learners’ age and proficiency level as well as to the content and genre of the writing task at hand” (Coyle and Roca De Larios, 2014, p453). Learners can use model texts to explore a variety of alternative words, structures, and ideas when comparing their own work to models (Hanaoka, 2007; Hanaoka and Izumi, 2012; Coyle and Roca De Larios, 2014). During this process, they are encouraged to intentionally recognize the language problems because mistakes are not overtly pointed out, which may promote more in-depth analysis (Adams, 2003; Sachs and Polio, 2007). Using model texts as a kind of feedback may have an impact on how well students digest writing assignments and maintain self-control since it mainly focuses on learning, remembering, and applying knowledge. It emphasizes learner initiative and shifts their participation from “mechanical to responsive” (Boud and Molloy, 2013). In addition, from a pedagogical perspective, since all of the students in the class are given the same model texts, a teacher’s workload is lessened compared with that of reformulating each student’s composition, which is time-consuming and less manageable (Yang and Zhang, 2010; Hanaoka and Izumi, 2012).

Despite model texts’ potential benefit in EFL or ESL writing, only a handful of empirical studies have examined the effectiveness of using model texts as a kind of WCF (Yang and Zhang, 2010; Cánovas Guirao et al., 2015; García Mayo and Labandibar, 2017; Coyle et al., 2018; Ramón, 2019; Coyle and Roca De Larios, 2020; Kang, 2020; Lazaro-Ibarrola, 2021; Luquin and Mayo, 2021; Kang, 2022). The prior research can be divided into two categories based on whether model texts were examined as the only WCF technique or were compared with other feedback strategies.

To the best of our knowledge, among the studies published on adopting model texts as the only feedback type, the initial effort was done by Hanaoka (2007), and the majority of subsequent research followed his research procedures and note-taking methods. 37 Japanese students engaged in a four-stage narrative picture description writing task. This research investigated the problems learners noticed while writing (Stage 1), how they compared their compositions against two model texts (Stage 2), and how such noticing influenced subsequent revisions in Stage 3 (immediate post-test) and Stage 4 (delayed post-test). The findings implied that when participants actively explored their difficulties, sought answers via model texts, and incorporated them into later revisions, they predominantly detected lexical features. Later, Martínez Esteban and Roca de Larios (2010) duplicated this research through employing a three-stage picture-based story writing task completed by 17 Spanish secondary school students. The findings indicated that students primarily identified lexical issues during the first stage and that they were unable to obtain adequate solutions to those difficulties in the model texts, most likely because the model texts had not been modified to their proficiency level. The participants incorporated a fair number of elements in their revisions, proving that models could be used as a kind of written feedback. However, since these studies did not include a control group, it remained uncertain whether the progress in participants’ writing was simply due to the model texts.

To resolve this methodological drawback, Cánovas Guirao et al. (2015) added a control group to examine the usefulness of adopting models in a three-stage picture description assignment with twenty 10-to-11-year-old children grouped into ten-sets of proficiency-matched pairs. The findings suggested that model texts were effective in drawing participants’ attention to lexical aspects and linguistic chunks instead of grammar. The degree to which feedback was noticed and acted upon was discovered to depend on language proficiency. After this research, the subsequent studies have included a control group, allowing the study to account for the effect of employing model texts as a kind of feedback. Similarly, García Mayo and Labandibar (2017) explored using model texts in a three-stage writing task with 60 teenage learners in the EFL context. The results showed that participants primarily identified their language gaps in the lexical aspects. Then, they were able to find solutions in the model texts and to integrate them into revision. In addition, learners with higher proficiency level and those who were guided during the process observed more features. Ramón (2019) is a study with 30 Spanish secondary students aged 13 and 14. The participants were divided into a Reporting Only Group (report the different features noticed) and a Reporting Plus Group (report the different features noticed, account for them and rehearse their use) and involved in a picture-based story writing assignment containing three phases. The results showed that the participants mainly focused on the aspects of lexis and ideas when compare their compositions with the model texts, and afterward during the final writing they were able to improve the aspects of lexis, form and ideas.

Unlike previous research, Kang (2020) utilized an argumentative writing task including four paragraphs to evaluate the effectiveness of using model texts as WCF in L2 writing. An analytic rubric was employed to grade participants’ writing. The participants were 40 high school students. Through analyzing participants’ writing performance in a three-phase writing assignment, the results showed that during the first writing phase and the comparison phase, participants were more aware of lexical issues, and model texts had been shown to have positive effect on boosting L2 learners’ lexical aspect and writing content. This research made the first attempt to employ a different writing type, and the findings indicated that the effects of model texts were influenced by the text type. Sixty seven college learners were used in Kang (2022) to determine if the working memory capabilities of students modify the impacts of model texts. The participants engaged in a three-phase writing assignment. The findings demonstrated that the participants who received model texts improved their writing significantly and kept these improvements 2 weeks later. Moreover, two key indicators of how much learners benefitted from using model texts were found to be complex working memory (assessing the central executive control system of L2 acquisition) and phonological short-term memory (measuring the phonological loop of L2 acquisition).

Though the above studies adopted similar approaches and yielded comparable results, some methodological issues should be considered in future studies. Firstly, the findings of these studies generally showed that participants were inclined to notice lexical aspects of their initial writing and model texts and, consequently, a substantial amount of lexis from model texts were integrated into their second writing or revision (Hanaoka, 2007; Cánovas Guirao et al., 2015; García Mayo and Labandibar, 2017). The specific writing type (picture-based story writing) used in these studies, however, can partially explain this result (Kang, 2020). The narrative picture-description writing task may prompt students to tell a story using some of the words provided on the pictures. This is likely to strengthen learners’ attention to vocabulary and then promote them to integrate more words from model texts into subsequent writing. Considering the effect of picture-based story writing tasks, it is necessary to employ diverse kinds of writing tasks. Besides, the model texts provided by previous research only included one short paragraph. Thus, it is difficult to investigate whether learners can pay attention to the macro-level features of language, such as the organization and coherence of several paragraphs. To further examine the effect of model texts of other writing types, the present research employs an argumentation assignment which requires students to compose several paragraphs. Secondly, most prior studies determined the usefulness of model texts by simply keeping track of the number of linguistic elements that were incorporated from models into subsequent writing (Hanaoka, 2007; Cánovas Guirao et al., 2015; García Mayo and Labandibar, 2017; Ramón, 2019). It is still a work in progress to figure out how to quantify writing improvement more precisely. Therefore, the present study analyzes the improvements of participants’ writing by using an analytic scoring rubric including four aspects of writing: lexis, grammar, content, and organization. Thirdly, it can be found that most studies investigated the effect of model texts by conducting a three-phase writing assignment: first writing, comparison, revision or rewriting (García Mayo and Labandibar, 2017; Ramón, 2019; Kang, 2020; Kang, 2022). One obvious drawback of the three-stage design is that the favorable impacts of models might be interpreted as proof of uptake rather than acquisition since the long-term effect of adopting models as WCF on learners’ writing performance is not clear. To enable scholars to examine learners’ language progress over longer time periods, it is necessary to use longitudinal research procedures. By means of adding a delayed post-test to a three-phase writing task, the present research further examines the long-term effect of adopting model texts as a WCF strategy in EFL writing.

Lastly, it can be seen from the review that previous studies have explored the role played by individual factors in applying model texts as a WCF strategy, such as learners’ language proficiency level (Cánovas Guirao et al., 2015; García Mayo and Labandibar, 2017), working memory capacity (Kang, 2022), and their attitudes towards modeling (García Mayo and Labandibar, 2017). It is crucial to explore the role played by other individual factors in using model texts as a feedback tool. To date, few studies have investigated the association between LAA (the capacity to analyze language by constructing and employing rules to generate sentences) and the efficacy of WCF (Shintani and Ellis, 2015; Benson and Dekeyser, 2019). These studies primarily monitored the mediating role of LAA on the effects of direct feedback and metalinguistic feedback. However, previous studies have failed to examine how LAA may influence EFL learners’ use of model texts as a kind of WCF. It is likely that LAA plays a crucial role in using model texts as a kind of WCF, considering that a model text is a form of feedback that requires learners to recognize, and generalize some language rules and patterns from it and engage in a process of reconstruction while comparing their own writing against model texts and composing a new composition. Therefore, the present study provides new insights into how LAA influences the effectiveness of using model texts and gives more information on the type of learner most likely to benefit from model texts.

Based on the above review, the current study filled these gaps by formulating the following research questions:

1. What aspects of language do EFL learners notice while writing an argumentative essay?
2. What aspects of language do EFL learners notice while comparing their compositions to model texts?
3. How do model texts improve EFL learners’ subsequent compositions?

## 3. Methodology

### 3.1. Contexts and participants

A comprehensive university in China was the setting of the present experiment. The participants were 60 Year 2 undergraduate students (39 females and 21 males) who have learned English for at least 10 years. The participants were matched in sets to higher LAA and lower LAA based on their LAA test scores, and then they were randomly assigned to three groups: (a) a control group (*n* = 20), which finished the writing, rewriting, and delayed writing phases without receiving model texts, (b) an unguided noticing (UN) group (*n* = 20), which completed all four stages (including writing, comparing, rewriting, and delayed writing), and employed an unguided noticing sheet when comparing own essays with model texts, (c) a guided noticing (GN) group (*n* = 20), which finished all four phases and employed a guided noticing sheet. Table 1 provides a summary of the data on participants.

**Table 1:** Participants in the study.

	Higher LAA level	Lower LAA level	Total number
Control group	10	10	20
Unguided noticing group	10	10	20
Guided noticing group	10	10	20
Total number	30	30	60

### 3.2. Research instruments

#### 3.2.1. Language analytic ability test

To measure their LAA, all participants were required to take an LAA test before the writing stage. The LAA test used in this research was based on Ottó’s language analysis exam used by Sheen (2007). This test has been frequently used to measure learners’ capacity to analyze language by constructing and employing rules to generate sentences. The test was made up of 14 multiple-choice questions. The participants were given a lexicon with English translations of words and sentences from an artificial language. Then, they were provided with 14 English sentences and required to select the right translation from four options for each sentence. The participants needed to analyze the grammatical markers provided in the glossary and applied them to the multiple-choice translations in order to select the proper choice.

**Figure 1:**
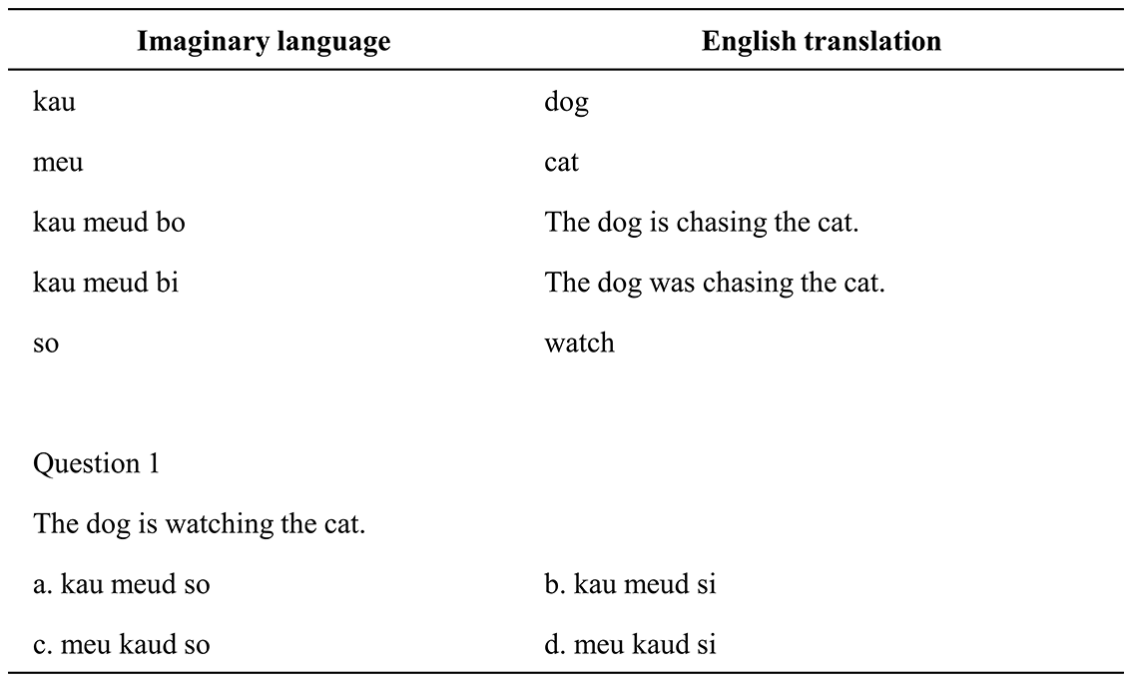
LAA test example.

Take the question in Figure 1 as an example. The participants must first infer the rule that “i” is a past progressive marker and “o” is a present progressive marker before applying it to the translated selections. The participants were required to complete the test within 15 min. Each correct answer in the test is worth one point, with a perfect score of 14 points. Given the exam’s complexity and the restricted testing time, the researcher guided participants through the first question, thereby reducing their anxiety and allowing them to become familiar with the exam. According to the scores of the participants (the average score is 9), those with a score of 9 or more are regarded as learners with higher LAA, and those with a score below 9 are regarded as learners with lower LAA.

#### 3.2.2. Writing tasks

Two argumentative writing tasks with similar topics were employed in the current work. The topic of the first writing task was “the importance of having a sense of social responsibility.” This composition was used in Stage 1 (writing) and Stage 3 (rewriting). The topic of the second writing task was “Why students should be encouraged to develop effective communication skills.” To reduce the impacts of task repetition, a different writing prompt was employed in the delayed writing. This step was intended to ensure that the new information the participants have stored and retrieved is not the result of repeatedly completing the identical task (Luquin and Mayo, 2021). Although the writing prompt was not the same, the participants can utilize and follow some expressions and patterns. These two writing tasks were chosen from previous College English Test Band 6 (CET-6) exam papers because all participants were going to take the CET-6 exam in the near future. Therefore, they were quite motivated to complete the writing tasks. The CET is a large-scale standardized test administered nationwide by the National College English Testing Committee on behalf of the Higher Education Department of the Ministry of Education in China. The CET-6 is administered twice a year. The purpose is to make scientific judgment of the comprehensive use of English for college students in China (Li and He, 2015; Liu and Zheng, 2022).

### 3.3. Research procedures

In Stage 1, the participants received Sheet 1 (see Supplementary Appendix A) to note down every problem they encountered during the first writing. They were required to write an essay of ranging between 150 and 200 words. They were asked to submit their compositions on pigaiwang,^1^ which can be used to collect and score students’ writing. Before submitting their writing, the participants were encouraged to proofread it to verify that any faults were not simple oversights that could be corrected by themselves. While writing, all participants were instructed to record their difficulties on Sheet 1 in English or Chinese. Although the researcher provided verbal instructions for participants before writing, specific instructions were given at the top of Sheet 1. Based on previous research (Hanaoka, 2007; García Mayo and Labandibar, 2017; Kang, 2020), Sheet 1 presented a list of examples of note-taking: “I do not know how to say/write X in English,” “I wrote X, but I am not sure if this is correct,” and “I do not know which tense to use when describing X,” etc. Specific examples were given according to the writing topic in order to reduce students’ cognitive loads. Because of class-time restrictions, the first phase had a 35-min time limit.

Immediately following Stage 1, the GN and UN group were given two model texts in Stage 2 and were required to note down any discrepancies between their texts and the two model texts. The UN and the GN groups employed different note-taking sheets. Two model texts were provided to participants to limit their chances of copying from a single model essay and enhance their opportunities to propose solutions to their difficulties (Coyle and Roca De Larios, 2014; García Mayo and Labandibar, 2017). Since the writing task originated from the CET-6 exam, this study collected model texts from two influential educational institutions that provide model essays for the CET-6 exam. Supplementary Appendix B presented complete versions of the two models. Following Hanaoka (2007), students in the UN group listed the differences they noticed in terms of several examples presented in Sheet 2 (see Supplementary Appendix C): “I did not know how to say/write X, but A/B uses Y,” “I have expressed the idea X this way, but A/B puts it this way,” and “I thought the past tense of the verb X was Y, but A/B writes Z.” As for the GN group, a note-taking table named Sheet 3 (see Supplementary Appendix C) was given to participants according to García Mayo and Labandibar (2017). While filling out this sheet, firstly, students had to figure out the kind of difference they noticed: lexis, grammar, content, organization, and others. Then, they were guided to write down the specific examples, and stated whether they approved the expressions in the model texts and why. For this part, examples were given at the top of Sheet 3 to help students express: “Yes. I did not know this English word,” “No, I think my expression was better than A/B,” and “Yes. I thought the tense used by A/B was correct.” This type of note-taking instruction was chosen to facilitate more in-depth noticing and processing. At the end of this stage, the two model texts and the participants’ note-taking sheets were collected by the researcher.

In Stage 3, each participant was asked to rewrite the same prompt. It took 15 to 20 min to complete the assignment. In order to prevent the participants from memorizing the models, they were not notified in advance of this writing task. One week after the third stage, they were required to complete the second argumentation “Why students should be encouraged to develop effective communication skills” (Stage 4). A time limit of 30 min was set for this fourth stage. This step aimed to figure out whether using models has long-term effect on EFL writing.

### 3.4. Data analysis

In this study, the primary data included: 1) 60 pieces of initial writing; 2) 60 note-taking sheets made in Stage 1; 3) 40 note-taking sheets made in Stage 2; 4) 60 pieces of rewriting in Stage 3; and 5) 60 pieces of delayed writing in Stage 4. SPSS 25 was used to perform quantitative analysis. The parametric tests in this study were performed on the basis of satisfying the assumptions of normal of distribution and homogeneity of variance. Through the qualitative analysis of participants’ notes and compositions, the researchers were able to extract the problems and difficulties that learners often encountered during the initial writing and identify the improvements or changes in participants’ subsequent writing.

For RQ 1 and 2, following Kang (2020), participants’ noticing in Stages 1 and 2 was recorded by note-taking which was divided into five types: lexis, grammar, content, organization, and others. The following are examples (originally written in Chinese) of each category based on the available data:

#### 3.4.1. Lexis

lexical terms that participants failed to seek out or spell in Stage 1 or previously unknown items that participants recognized in Stage 2:

1. *I cannot spell ‘zhunze’ in English* (Stage 1).
2. *I wrote ‘company’ but the model text used ‘enterprise’* (Stage 2).

#### 3.4.2. Grammar

Features that emphasize tense usage and preposition selection:

3. *I am unsure whether to use ‘be harmful to’ or ‘be harmful at’* (Stage 1).
4. *I used future tense in many sentences, but the model texts mainly employed simple present tense* (Stage 2).

#### 3.4.3. Content

Features associated with the generation, development or expression of ideas:

5. *I could not think of any examples to support my argument* (Stage 1).
6. *The model text and I provided evidence from different perspectives to support the same type of opinion* (Stage 2).

#### 3.4.4. Organization

Features related to the structure or coherence of composition:

7. *Do I have to indent the first line of every paragraph?* (Stage 1).
8. *I wrote a title for my composition while the model text did not include a title* (Stage 2).

#### 3.4.5. Other

Features which did not belong to the above four types:

9. Time for *composition* writing is a bit tight for me.

For RQ 3, participants’ first writing, subsequent rewriting, and delayed writing were scored in the light of the analytical scoring rubric employed in Kang (2020) (see Supplementary Appendix D). Like the coding method of note-taking, the compositions were assessed from the following aspects: lexis, grammar, content, and organization. The highest and lowest scores for each aspect were 3 and 0, respectively. The maximum score was twelve. The scores of each aspect of writing were then added together to generate a global score. In this way, the researchers were able to specify which areas of writing were enhanced as a consequence of reading model texts. As for the rating process, two raters first evaluated three compositions together to standardize the rating criterion. Then, they scored the remaining compositions individually. The interrater reliability was 95%.

## 4. Results

### 4.1. The language features noticed by EFL learners during the initial writing stage

RQ 1 centered on the categories of difficulties that participants might encounter throughout their first argumentative writing task. Table 2 presents that with an average of 5.58 problems per participant, the participants identified 335 language problems in total. In addition, more than half of the features were lexical (60.6%), indicating that the participants focused most on looking for appropriate lexis to convey their views in the initial writing stage.

**Table 2:** Frequencies and proportions of problems noticed in Stage 1.

	All participants (*N* = 60)				Higher LAA group (*N* = 30)				Lower LAA group (*N* = 30)			
	*n*	%	Mean	SD	*n*	%	Mean	SD	*n*	%	Mean	SD
Lexis	203	60.6	3.38	1.54	98	60.12	3.27	1.36	105	61.05	3.5	1.72
Grammar	29	8.66	0.48	0.65	13	7.98	0.43	0.63	16	9.3	0.53	0.68
Content	80	23.88	1.33	0.97	39	23.93	1.3	0.88	41	23.84	1.37	1.07
Organization	16	4.78	0.27	0.48	10	6.13	0.33	0.55	6	3.49	0.2	0.41
Other	7	2.09	0.12	0.32	3	1.84	0.1	0.31	4	2.33	0.13	0.35
Total	335	100	5.58	1.86	163	100	5.43	1.85	172	100	5.73	1.89

Considering the effects of LAA on noticing, no significant differences were found between the higher level LAA participants and the lower level LAA participants in the total amount of problems noticed (*t* = 0.621, df = 58, *p* = 0.537). Levene’s test was not significant (*p* > 0.05); therefore, the assumption of homogeneity of variances was tenable. Moreover, a MANOVA test was performed to find out the impact of LAA on different category of language issue. The assumption of homogeneity of variance was met (Box’s test was not significant at *p* > 0.001), and the assumption of equality of variance for dependent variables was tenable (Levene’s test was not significant at *p* > 0.05). No significance was evident (Wilks’s lambda test statistics = 0.965, *F*_(4,55)_ = 0.500, *p* = 0.735).

Further analysis was conducted to see whether the three groups at both LAA level differed significantly from one another. The frequency of problems observed was compared across the different groups using one-way ANOVA. The assumption of homogeneity of variances was met since Levene’s test was not significant (*p* > 0.05). No differences between the higher LAA level (*F*_(2,27)_ = 0.971, *p* = 0.392) and the lower LAA level (F_(2,27)_ = 1.417, *p* = 0.260) were found to be statistically significant. The categories of problems noted by the various groups at each LAA level were further examined using MANOVA tests to determine whether there was any statistically significant difference. No serious violations of the assumption of homogeneity of variance (Box’s test was not significant at *p* > 0.001) and the assumption of equality of variance for dependent variables (Levene’s test was not significant at *p* > 0.05) were detected. No significances were found in the higher LAA level participants (Wilks’s lambda test statistics = 0.820, *F*_(8,48)_ = 0.625, *p* = 0.753) and the lower LAA level participants (Wilks’s lambda test statistics = 0.806, *F*_(8,48)_ = 0.685, *p* = 0.703). These findings reveal that participants in the three groups at each LAA level noticed comparable issues during the early stage.

### 4.2. The language features noticed by EFL learners during the comparison stage

RQ 2 explored the items that participants noticed when comparing their own writing to model texts and if there were any differences between the UN group and the GN group. As shown in Table 3, the participants recorded 310 features in all, with an average of 7.75 features per person. Similar to Stage 1, the participants mainly noticed lexical issues (71.94%) in Stage 2.

**Table 3:** Frequencies and proportions of features in Stage 2.

	All participants (*N* = 40)				Higher LAA group (*N* = 20)				Lower LAA group (*N* = 20)			
	*n*	%	Mean	SD	*n*	%	Mean	SD	*n*	%	Mean	SD
Lexis	223	71.94	5.57	3.18	133	74.72	6.65	3.1	90	68.18	4.5	2.95
Grammar	26	8.39	0.65	0.98	12	6.74	0.6	0.94	14	10.61	0.7	1.03
Content	38	12.26	0.95	0.85	21	11.8	1.05	0.89	17	12.88	0.85	0.81
Organization	21	6.77	0.53	0.78	11	6.18	0.55	0.76	10	7.58	0.5	0.83
Other	2	0.65	0.05	0.22	1	0.56	0.05	0.22	1	0.76	0.05	0.22
Total	310	100	7.75	3.7	178	100	8.9	3.78	132	100	6.6	3.32

Considering the effects of LAA on the total amount of items found in the second phase, the participants with higher LAA significantly detected more features than the participants with lower LAA (*t* = 2.05, df = 38, *p* = 0.042). The assumption of homogeneity of variances was not violated since Levene’s test was not significant (*p* > 0.05). Similarly, with no serious violations of assumptions, a MANOVA test was performed to examine the impact of LAA on each type of language feature that was noted in the comparison stage. The assumption of homogeneity of variance was met (Box’s test was not significant at *p* > 0.001), and the assumption of equality of variance for dependent variables was tenable (Levene’s test was not significant at *p* > 0.05). No significance was evident on the combined dependent variables (Wilks’s lambda test statistics = 0.878, *F*_(4,35)_ = 1.219, *p* = 0.320). When the findings for the dependent variables were taken into separate consideration, only the lexical features noted by the higher level LAA participants and the lower level LAA participants differed enough to be statistically significant (*F*_(1,38)_ = 5.054, *p* = 0.030).

Additionally, a comparison of the GN group and the UN group’s data was done to look for any significant differences at each LAA level. The total amount of features observed by the GN group and the UN group were compared using two *t* tests to access if there were any significant differences. Significant differences were found in the overall amount of items noted by GN group and UN group at both higher LAA level (*t* = 6.593, df = 18, *p* < 0.001) and lower LAA level (*t* = 2.224, df = 18, *p* = 0.039). The assumption of homogeneity of variances was tenable since Levene’s test was not significant (*p* > 0.05). Then, the categories of problems identified by the two groups at different LAA levels were compared using MANOVA test to see whether there were any statistically significant differences. The preliminary analyzes indicated no serious violation of the assumption of homogeneity of variance (Box’s test was not significant at *p* > 0.001) and the assumption of equality of variance for dependent variables (Levene’s test was not significant at *p* > 0.05). The tests showed that with comparison to the UN group, the GN group with higher LAA concentrated significantly more on lexis (*F*_(1,18)_ = 15.359, *p* = 0.001) and organization (*F*_(1,18)_ = 10.565, *p* = 0.004), while the GN group with lower LAA concentrated significantly more on organization (*F*_(1,18)_ = 11.250, p = 0.004).

### 4.3. The effects of model texts on EFL learners’ subsequent compositions

In response to the third research question, this study compared participants’ initial writing in Stage 1(pre-test), rewriting in Stage 3 (immediate post-test), and delayed writing in Stage 4 (delayed post-test). These compositions were graded by using the analytical scoring rubric (Supplementary Appendix D). Table 4 shows the descriptive statistics of the writing tasks.

**Table 4:** Descriptive statistics for the three compositions.

	Control group (*N* = 20)						Unguided noticing group (*N* = 20)						Guided noticing group (*N* = 20)					
	Pre		Post		Delay		Pre		Post		Delay		Pre		Post		Delay	
	M	SD	M	SD	M	SD	M	SD	M	SD	M	SD	M	SD	M	SD	M	SD
Vocabulary	1.35	0.49	1.4	0.5	1.3	0.47	1.35	0.49	1.75	0.55	1.6	0.5	1.35	0.49	2.15	0.37	2.1	0.45
Grammar	1.25	0.44	1.3	0.47	1.35	0.49	1.25	0.44	1.3	0.47	1.85	0.67	1.3	0.47	1.95	0.76	2.2	0.52
Content	1.45	0.51	1.5	0.51	1.5	0.51	1.5	0.51	2	0.65	1.8	0.41	1.5	0.51	2.3	0.66	2.15	0.49
Organization	1.75	0.44	1.75	0.44	1.75	0.44	1.85	0.37	2.2	0.52	2	0.32	1.6	0.5	2.5	0.51	2.4	0.5
Total	5.8	1.32	5.95	1.32	5.9	1.12	5.95	0.83	7.25	1.16	7.25	0.85	5.75	1.16	8.9	1.59	8.85	1.39

Before running the test, the assumption of homogeneity of variances was examined. Levene’s test was not significant (*p* > 0.05); therefore, the assumption was not violated. In terms of the overall score on the pre-test, one-way ANOVA was used to see whether the three treatment groups were statistically different before the experiment began. There was no significant difference between the three groups (*F*_(2,57)_ = 0.172, *p* = 0.843). Repeated-measures ANOVAs were employed to check whether using model texts significantly influence the overall performance and different aspects of participants’ subsequent writing and delayed writing. The assumption of homogeneity of variance (Box’s test was not significant at p > 0.001) and the assumption of equality of variance for variables (Levene’s test was not significant at p > 0.05) were met. The findings presented that there was a significant effect for the different treatment on the participants’ overall performance of the writing tasks (*F*_(4,114)_ = 28.479, *p* < 0.001). There were significant differences in the vocabulary (*F*_(4,114)_ = 10.691, *p* < 0.001), grammar (*F*_(4,114)_ = 7.826, *p* < 0.001), content (*F*_(4,114)_ = 5.292, *p* = 0.001), and organization (*F*_(4,114)_ = 8.067, *p* < 0.001) aspects.

Then, further investigation was done by running one-way ANOVAs for the immediate rewriting and delayed writing to see which group did best, and whether the two treatment groups were different from the control group. The descriptive statistics revealed that in both post-tests the GN group scored the highest and the control group scored the lowest. With no violation of the assumption of homogeneity of variances (Levene’s test was not significant at *p* > 0.05), results for the immediate post-test (*F*_(2,57)_ = 23.398, p < 0.001) and delayed post-test (*F*_(2,57)_ = 33.551, p < 0.001) were both statistical. The findings of the immediate post-test suggested that both the GN group and the UN group had significant better performance than the control group, and the GN group was different from the UN group. The numbers for the delayed post-test were slightly different but the overall trend was consistent, with the GN group statistically better than all others, but both experimental groups performing better than the control group.

## 5. Discussion

The primary aim of this work was to elucidate the language items EFL learners noticed at the first writing stage and the comparing process, explore the effect of LAA on participants’ noticing and use of model texts, and investigate the effectiveness of adopting model texts as a WCF strategy to improve writing performance.

### 5.1. The language features noticed by EFL learners during the first writing stage

For RQ 1, considering the effect of LAA on noticing during the initial writing, the data showed that learners with lower LAA noticed more linguistic problems than those with higher LAA, but the difference did not reach significance. The findings revealed that participants chiefly reported lexical issues while completing the compositions. This lexical-oriented noticing is in line with previous studies (Hanaoka, 2007; Coyle and Roca De Larios, 2014; Cánovas Guirao et al., 2015; García Mayo and Labandibar, 2017; Kang, 2020). Such a focus on lexis may be due to learners’ limited processing capacity, which leads them to look for content-related items and grammatical issues when they have a surplus energy for noticing (Vanpatten, 1996). In this study, however, the number of lexical problems noted in the first writing stage accounted for 60.6% of the total, which is less than the number presented in previous studies but quite similar to Kang (2020). This finding supports Kang’s (2020) claim that this difference may be attributed to the genre of writing tasks that were assigned. With the exception of Kang (2020), prior research primarily employed picture-based narrative writing tasks in which learners needed to think of appropriate words to describe the picture prompts. This type of writing task might have brought learners’ lexical deficits to the forefront of their minds. Nonetheless, the argumentative tasks adopted in the current study demanded learners to not only recollect pertinent words, but also construct their arguments and present them in a logical order that made sense to the reader. Thus, the participants in this study expanded their noticing beyond lexis to include grammar, content, and organization.

By examining the participants’ notes from the initial writing stage, the researchers discovered that the linguistic problems encountered by the participants shared some similarities. For the lexical problems noticed by the participants, this study revealed that the lexical issues the participants noted were closely related to the writing topic——social responsibility. Some of these words were mentioned frequently, such as “harmonious,” “obligation” or “duty,” “criterion” or “norm,” “morality,” “individual,” “cultivate.” Besides, some participants claimed that there were not enough synonyms for them to select, and they believed that the words they had used in their compositions did not belong to advanced vocabulary. This situation indicates that some participants do not have sufficient vocabulary accumulation and their exposure to authentic English expressions is lacking. Besides, the feeling of “lacking advanced vocabulary” is a sign of low self-confidence in EFL writing.

Regarding the grammatical difficulties noted by the participants, they were mainly confused of which tense to use when discussing certain social phenomena or pointing out the importance of improving people’s sense of social responsibility. For example, student 1 in the control group wrote that *I am not sure what tense I should use to express the line ‘Egoism is becoming increasingly prevalent’*; student 2 in the GN group noted that *I do not know which tense to use to describe the line “Only those who have a sense of social responsibility will be loved and respected by others*.*”*

Concerning the content problems identified by the participants, almost all of those who reported their problems had the same difficulties of lacking specific examples or being unable to recall relevant instances or phenomena. In addition, a few participants stated that the content of their compositions was vague and hollow.

When it comes to the organizational issues presented by the participants, this research found that the participants faced a number of similar problems. For instance, several participants were unclear about how many paragraphs should be included in this argumentative writing task and how to allocate the proportion of each paragraph. Moreover, some of the participants pointed out that their essays were not very logically consistent, and the division of paragraphs was ambiguous. As for the other problems noticed by the participants, they mainly focused on the time limitation and the word count requirement of the writing task. Several participants complained that they did not have enough time or words to write to complete the writing task.

Moreover, it is essential to highlight that the participants in the current research reported 8.66% grammatical problems and 23.88% content-related problems which largely exceeded these two kinds of language problems reported by previous studies (e.g., Hanaoka, 2007; Coyle and Roca De Larios, 2014; García Mayo and Labandibar, 2017). The genre of writing assignment and the participants’ ages appear to be associated with the increased attention to grammatical and content-related aspects observed in the current study. Though prior research basically utilized narrative picture-description writing and young learners such as children and teenage learners (e.g., Coyle and Roca De Larios, 2014; García Mayo and Labandibar, 2017), this study employed the argumentation assignment and focused on college EFL learners. It is likely that the adult learners are more conscious of grammatical rules and are creative and productive in thinking and expressing their own ideas since they have higher cognitive capacities and foreign language ability. Therefore, the participants might be able to notice and note down more problems in the aspects of grammar and content at the first writing phase. Additionally, the present research was carried out in an environment that placed more emphasis on proper grammar and precise language use. Compared to learners in a setting where instruction is meaning-oriented, L2 learners in such circumstances are inclined to exhibit a higher level of grammatical form awareness (Sheen, 2004). Thus, this finding further implies that the genre of writing task, the age of participants, and the instructional setting may influence how learners allocate their attention when writing.

Based on the above analysis, several suggestions can be put forward to assist teachers in lessening learners’ cognitive burden before writing practices. For example, to reduce the lexical problems the learners may encounter, teachers can provide a list of keywords associated with the topic before writing. Teachers can also conduct brainstorming sessions and lead learners to discuss relevant examples to enrich their writing content. Furthermore, teachers can explain how to organize the beginning, body, and conclusion part of argumentative writing to help learners build a writing framework.

### 5.2. The language features noticed by EFL learners during the comparison stage

For the second research question, the lexical-oriented noticing (71.94%) is consistent with previous research which reported similar results (Hanaoka, 2007; Cánovas Guirao et al., 2015; García Mayo and Labandibar, 2017; Kang, 2020). Such a lexical-oriented noticing could be the result of the “priming” effect of output (Izumi, 2003). This effect prompted the learners to search for relevant vocabulary in the model texts in order to fill the gaps they identified during writing. It indicates that through output, learners might primarily be aware of their “lexical holes” (Swain, 1998) in the interlanguage. This perception of a need for vocabulary resulted in searching for lexis-related solutions in the two model texts. Lexis, especially in note-taking conditions, is easy to express, describe, and remember. It is also a possibility that the perceived need for lexis is more pronounced under high physical demands. Additionally, through analyzing the participants’ notes at the first writing stage, it can be found that the majority of participants believed that the lexis they had used in their compositions did not belong to advanced lexis. This might promote them to look for some “advanced lexis” in the two model texts and record them on the note-taking sheets. Another finding that should be mentioned is that the participants could recognize new gaps in initial compositions and, as a result, paid more attention to the expression, content, and organization of the texts during the comparing process. These findings are consistent with Hanaoka (2007), Martínez Esteban and Roca de Larios (2010), and García Mayo and Labandibar (2017), and support the positive function that model texts play in encouraging the participants to notice new features and in broadening their range of concerns.

The data showed that the participants with higher LAA noticed significantly more items during the comparing process than the participants with lower LAA. While reading model texts, participants tried to decipher the meaning of feedback as they strived to comprehend its worth and make sense of the information it contains. In previous research that considered the effect of language proficiency, the findings indicated that more proficient learners noticed more language-related features (Hanaoka, 2007; García Mayo and Labandibar, 2017). Therefore, it is possible to conclude that the learners with higher LAA could extract more information from the model texts and were more likely to identify distinctions between their own compositions and the models. In addition, the results showed that the GN group at the higher LAA level focused significantly more on lexis and organization, while the GN group at the lower LAA level focused significantly more on organization. This finding implies that, under the same guidance, the participants with higher LAA seem to have a broader range of attention while comparing their compositions and model texts.

The GN group noticed more features than the UN group, and this situation is applicable in both high LAA level and low LAA level. In addition, taking notes is a very effective way to guide learners to understand the model texts. Especially in the case of guided note-taking, learners can get help to better pay attention to the two model texts. This finding is in line with García Mayo and Labandibar (2017) who found that the GN groups at both proficiency levels noted more features while comparing their output with model texts, whereas the difference in their study did not reach significance. Therefore, the current research further supports their point of view that there seems to be a correlation between guidance and improved noticing.

Regarding the specific features the participants noticed during the comparison stage, this research found that many of the problems raised by the participants at the first stage have been solved by reading the model texts, and some problems that they did not report at the beginning were also resolved. This included not only the lexical problems that the participants focused on the most, but also other aspects like grammar, content, and organization. For instance, as regards the lexical issues solved at this stage, most of them belonged to the keywords closely related to the writing topic, such as “morality,” “harmonious,” and “prevalent.” The grammatical issues solved at the comparison stage were mainly the correct tense that should be used while introducing social phenomena and emphasizing the significance of having a sense of social responsibility. Regarding the content problems solved at this stage, most participants noted that from reading models, they could determine which examples could be adopted to support the writing’s argument. As for the organizational problems addressed during comparison, both model texts consisted of three paragraphs, with the first sentence of each paragraph indented. All these information gained from reading the model texts were helpful in solving the problems encountered by the participants. Therefore, the participants who received model texts performed better in the rewriting stage.

Moreover, it should be highlighted that the majority of participants also recorded a number of elements they deemed instructive in the model texts, and some of these elements of various aspects of writing were repeatedly mentioned by the participants. For example, among the most impressive words they noticed, words like “imperative,” “pillar,” “ascribe” and “violation” were frequently referred to by the participants, and they regarded these words as the “advanced vocabulary” that they cannot come up with in the first draft. These words can be employed not only for the first writing assignment, but also for other argumentative writing tasks. Thus, it is likely that providing model texts to learners alerts them to accumulate some advanced words that are commonly used in the same type of writing. Concerning the grammatical features repeatedly cited, the participants mainly focused on the use of inverted sentence in the model texts. As for the content features, most of the participants shared similar opinions that the two model texts were informative, and the examples presented by them were quite pertinent to the writing topic. Besides, a lot of participants noted that model text A highlights the importance of having a sense of social responsibility from the standpoint of the company and the individual, whereas model text B utilizes counterexamples to prove the argument. It can be seen that the participants were capable of summarizing the central content of model texts.

In general, compared with traditional error correction feedback, the use of model texts gives learners a wide range of alternatives and may encourage them to engage in more in-depth processing.

### 5.3. The effects of model texts on EFL learners’ subsequent compositions

RQ 3 investigated how much the learners’ writing can be improved by the use of model texts. The results showed that, in general, the participants who received model texts were able to enhance their subsequent compositions by improving expressions and grammatical accuracy, enriching content, and making structure more coherent and logical. This was also proved by the fact that their total writing scores were significantly higher than those of the control group.

Apart from receiving a better total score, the participants who were given models outperformed those in the control group in terms of vocabulary, grammar, content, and organization. During the rewriting stage, a number of convincing statements and pertinent examples are used by students to back up their arguments. These arguments often come from the model texts that learners received. The model texts help students who start writing without a clue, allowing them to have more supporting information to illustrate their arguments. The higher writing scores of two treatment groups validates the beneficial effects of model texts discovered in prior studies (Hanaoka, 2007; Cánovas Guirao et al., 2015; García Mayo and Labandibar, 2017; Kang, 2020).

Through observing the notes and compositions, this study found that what the participants noticed during the initial writing strongly influenced what they focused on during the comparing stage. In the same way, the integration and retention of alternatives in subsequent writing may also be predicted by this effect. These findings confirm the data reported by Hanaoka (2007) and García Mayo and Labandibar (2017). This appears to suggest that output creates a willingness in learners to explore solutions and also allows them to identify gaps. Besides, output has a positive effect on enhancing learners’ motivation to integrate existing solutions. Moreover, it should be mentioned that the participants who were given model texts integrated other features that they did not perceive as problematic while completing writing. This may suggest that compared with other error correction strategies, model texts are beneficial for learners to find alternative ways to express and organize their ideas.

In the two subsequent writing tasks, the difference between the GN and UN groups significantly illustrated the effect of guidance on the inclusion of model texts. Consistent with the findings of Qi and Lapkin (2001), the better-performing GN group was able to engage in deeper levels of comprehension. This demonstrates that greater intake, aided by guided noticing, is associated with the noticing with comprehension. However, to obtain long-term memory, rehearsal is essential even in the case of noticing with comprehension (Hanaoka, 2007; Sachs and Polio, 2007). Their statements demonstrated the significance of developing extra activities and practices with the intention of reinforcing the linguistic features noticed. Besides, even though the participants picked up new words and expressions from the model texts, some of them might not have recognized the model texts as a kind of WCF strategy because using model texts is an implicit technique of offering corrections. Therefore, in order to optimize the benefits of model texts, teachers should illustrate how to utilize model texts to develop the learners’ writing and provide supplementary guidance on the language features or writing skills included in the model texts.

## 6. Conclusion

The current research examined the usage of model texts as positive evidence feedback in a four-phase argumentation assignment completed by 60 EFL college learners. The results showed that using model texts could be beneficial for helping learners to focus on various components of essay like lexis, grammar, content, and organization. While reading model texts, learners could identify some solutions to their linguistic problems and other linguistic features and then incorporate them in their second and delayed compositions. Adopting model texts is helpful in enhancing learners’ writing performance, especially the vocabulary, content, and organization aspects. Moreover, the data indicated that higher LAA learners could obtain more knowledge from the model texts and were more likely to recognize differences between the models and their writing. The guidance of note-taking could promote learners’ noticing and engage them in deeper understanding, which strengthened the incorporation of model texts to improve their writing performance.

Based on these findings, some pedagogical implications can be made. In light of the situation that enhancing the depth of noticing is essential for increasing the integration and preservation of linguistic items, it is necessary to give students extra activities and practices on the way of taking notice of various aspects of writing and employing model texts. For example, teachers can organize pair discussions, group discussions, or even class discussions to address the problems that learners are not clear about. By reducing the workload associated with writing tasks, this teamwork might encourage learners to have more positive attitudes towards model texts and subsequent writing. Besides, teachers could directly instruct learners to observe how expressions and forms are used in the model texts, how ideas are conveyed to illustrate the topic, and how arguments are arranged to be logical and coherent. When learners share expectations and comprehension with their teacher concerning the usefulness and rationale behind the feedback (Lee et al., 2021), they are more inclined to take additional effort to process and employ it to enhance their writing (Zhang, 2022). Therefore, it is essential to foster learner engagement with model texts by constructing and extending mutual understanding between learners and teachers.

However, this paper has certain limitations that need to be noted. First, this study only employed note-taking to examine what kind of features the participants noticed. Due to the time-consuming and laborious nature of note-taking, participants may not take detailed notes on everything they observe. Second, the present study employed a delayed post-test that was conducted 1 week after the initial draft. But the comparability between the initial writing topic and the topic used in the delayed post-test wasn’t estimated through a pilot study. Third, this study only employed the genre of argumentative writing to examine the usefulness of model texts. At last, this study only examined the effect of LAA on learners’ use of model texts in EFL writing.

Based on these limitations, several suggestions are put forward for future studies. Firstly, future research could examine participants’ noticing by using other verbalization methods like think-aloud and stimulated recall. Secondly, it is worthwhile to further explore how long the positive effects may persist. Future studies should employ longitudinal designs that help scholars to assess learners’ linguistic development after 1 week. Additionally, it is necessary to estimate how comparable the initial writing topic and the topic used in the delayed post-test are through a pilot study. Thirdly, future studies should examine the effects of model texts in EFL writing with other genres of writing, such as expository writing and letter writing. Finally, future research on how other individual factors (such as learners’ beliefs, motivation) influence the process of utilizing model texts may shed light on which kind of learners gains more through receiving model texts.

## Data availability statement

The raw data supporting the conclusions of this article will be made available by the authors, without undue reservation.

## Ethics statement

The studies involving human participants were reviewed and approved by Southeast University. Written informed consent to participate in this study was provided by the participants.

## Author contributions

All authors listed have made a substantial, direct, and intellectual contribution to the work and approved it for publication.

## Funding

This manuscript was supported by the Special Research Project of “Jointly Building a New Ecology of High-quality Foreign Language Education” in Jiangsu Universities (Project No.: 2022WJYB002).

## Conflict of interest

The authors declare that the research was conducted in the absence of any commercial or financial relationships that could be construed as a potential conflict of interest.

## Publisher’s note

All claims expressed in this article are solely those of the authors and do not necessarily represent those of their affiliated organizations, or those of the publisher, the editors and the reviewers. Any product that may be evaluated in this article, or claim that may be made by its manufacturer, is not guaranteed or endorsed by the publisher.

## Footnotes

^1^ www.pigai.org
