# Supplementary material for: Using model texts as a type of feedback in EFL writing
Source: Front Psychol. 2023 Jun 29;14:1156553. doi: 10.3389/fpsyg.2023.1156553 (PMC10344447; doi:10.3389/fpsyg.2023.1156553)
Supplement: Supplementary file 1 [file Data_Sheet_1.DOCX]

***Supplementary Material***

**Using model texts as a type of feedback in EFL writing**

**Zhixin Wu, Jiaxin Qie*, Xuehua Wang**

*** Correspondence:** Jiaxin Qie: qiejiaxin0619@163.com

**Appendix A. Sheet 1 for Stage 1**

Please note down any difficulties or doubts you had while writing the composition. You can use the following expressions if you find them useful, but you can also use your own words:

- *I do not know how to say/write ______ in English.*
- *I wrote ______, but I am not sure if this is correct.*
- *I do not know which tense to use when describing ______.*
- *The word ______ was written in the past tense, but I am not sure if this is correct.*
- *I cannot think of any examples that are related to the topic.*
- *I can only think of one or two examples, which is not enough to support my argument.*

**Appendix B. Two model tests used in the study**

*[Model text A]*

Problems such as the violation of privacy and environmental pollution are becoming increasingly prevalent in modern society. To some extent, they can be ascribed to a distinct lack of a sense of social responsibility among enterprises and individuals, which is of crucial importance in building a better world.

When seeking profits, socially responsible corporations never forget to make positive contributions to our society. Take Alibaba, a leading tech company in China as an example, the Ant Forest green initiative it launched in 2016 has so far inspired up to 500 million Alipay users to adopt low-carbon lifestyles and resulted in over 100 million trees planted in China’s most barren regions. At the individual level, a sense of social responsibility motivates us college students to acquire as much knowledge as possible and put it into practice. It also prevents us from engaging in unethical behaviors such as campus violence and academic misconduct that cause negative social impacts.

To sum up, a healthy society requires all its members to take on their respective responsibilities. Everyone, therefore, should develop a sense of social responsibility, especially we college students who will be a pillar of society in the future.

*[Model text B]*

There is no doubt in saying that having a strong sense of responsibility is of great significance for social harmony, which should not be ignored by the young generation.

However, with the increasingly fierce competition in modern society, a growing number of youngsters tend to be under tremendous pressure. As a result, they are more likely to focus on their own short-term interests, ignoring the value of social responsibility. A case in point is the decline of social ethics and morality. As a matter of fact, nowadays, a number of citizens are not willing to help others who are in need of help and indifferent to their suffering. If we left the current situation alone, hardly could we live in a harmonious and loving society.

In conclusion, it is imperative to take some corresponding measures. For one thing, the mass media should enhance the publicity, enlightening the young as to cultivating a strong sense of social responsibility. For another, it is advisable that individuals should pay more attention to what will benefit us all in the future.

**Appendix C. Sheet 2 and 3 for Stage 2**

**Sheet 2: Unguided noticing group**

Please compare your composition with the two model texts provided (A and B) and note down all the differences that you find. You can use the following expressions if you find them useful, but you can also use your own words:

- *I did not know how to say/write the word ______, but A/B uses ______.*
- *I have expressed the idea ______ this way, but A/B puts it this way ______.*
- *I was impressed by the expression of ______ in A/B.*
- *I thought the past tense of the verb ______ was ______, but A/B writes ______.*
- *I use ______ tense while describing ______, but A/B uses ______.*

**Sheet 3: Guided noticing group**

Please compare your composition with the two models provided (A and B) and note down all the differences that you find following the table below.

| Type | Difference | Do you think the model’s alternative is more appropriate? Why? |
| --- | --- | --- |
| Lexis | - *I did not know how to say/write the word ______, but A/B uses ______.* - *I have expressed the idea ______ this way, but A/B puts it this way ______.* - *I was impressed by the expression of ______ in A/B.* - *Other:* | *Yes. I did not know this English word. / No, I think my expression was better than A/B.* |
| Grammar | - *I use ______ tense while describing ______, but A/B uses ______.* - *I thought the past tense of the verb ______ was ______, but A/B writes ______.* - *Other:* | *Yes. I thought the tense used by A/B was correct.* |
| Content | - *I provided ______ example(s) to support my argument, but A/B provides ______ example(s).* - *Other:* |  |
| Organization | - *I did not indent the first sentence of each paragraph, but A/B has.* - *Other:* |  |
| Other |  |  |


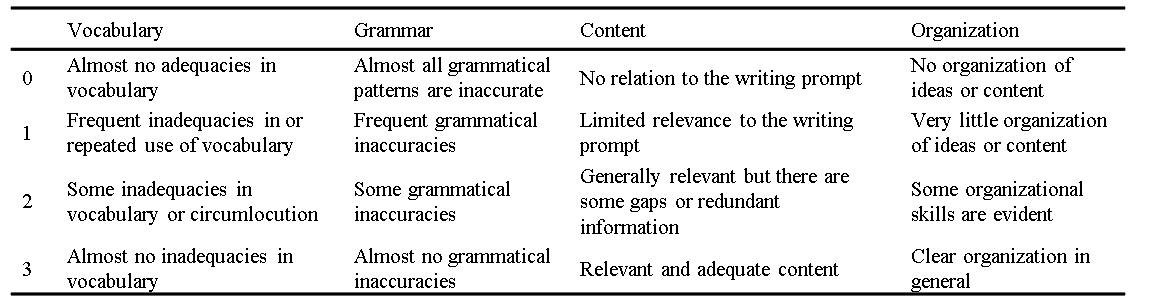
**Appendix D. Scoring rubric**
